# Technical report: liquid overlay technique allows the generation of homogeneous osteosarcoma, glioblastoma, lung and prostate adenocarcinoma spheroids that can be used for drug cytotoxicity measurements

**DOI:** 10.3389/fbioe.2023.1260049

**Published:** 2023-10-06

**Authors:** Camille Jubelin, Javier Muñoz-Garcia, Denis Cochonneau, Emilie Ollivier, François Vallette, Marie-Françoise Heymann, Lisa Oliver, Dominique Heymann

**Affiliations:** ^1^ Nantes Université, Centre National de la Recherche Scientifique (CNRS), Unit in Biological Sciences and Biotechnologies (US2B), Nantes, France; ^2^ Institut de Cancérologie de l’Ouest, Tumor Heterogeneity and Precision Medicine Lab, Saint-Herblain, France; ^3^ Atlantic Bone Screen, Saint-Herblain, France; ^4^ Nantes Université, Institut National de la Santé et de la Recherche Médicale (INSERM), Centre de Recherche en Cancérologie et Immunologie Nantes-Angers (CRCI^2^NA), Nantes, France; ^5^ CHU de Nantes, Nantes, France; ^6^ Department of Oncology and Metabolism, Medical School, University of Sheffield, Sheffield, United Kingdom

**Keywords:** 3D culture (three-dimensional spheroids), homogeneous spheroid model, liquid overlay technique, hanging drop, lung adenocarcinoma, prostate adenocarcinoma, osteosarcoma, glioblastoma

## Abstract

**Introduction:** The mechanisms involved in cancer initiation, progression, drug resistance, and disease recurrence are traditionally investigated through *in vitro* adherent monolayer (2D) cell models. However, solid malignant tumor growth is characterized by progression in three dimensions (3D), and an increasing amount of evidence suggests that 3D culture models, such as spheroids, are suitable for mimicking cancer development. The aim of this report was to reaffirm the relevance of simpler 3D culture methods to produce highly reproducible spheroids, especially in the context of drug cytotoxicity measurements.

**Methods:** Human A549 lung adenocarcinoma, LnCaP prostate adenocarcinoma, MNNG/HOS osteosarcoma and U251 glioblastoma cell lines were grown into spheroids for 20 days using either Liquid Overlay Technique (LOT) or Hanging Drop (HD) in various culture plates. Their morphology was examined by microscopy. Sensitivity to doxorubicin was compared between MNNG/HOS cells grown in 2D and 3D.

**Results:** For all cell lines studied, the morphology of spheroids generated in round-bottom multiwell plates was more repeatable than that of those generated in flat-bottom multiwell plates. HD had no significant advantage over LOT when the spheroids were cultured in round-bottom plates. Finally, the IC_50_ of doxorubicin on MNNG/HOS cultured in 3D was 18.8 times higher than in 2D cultures (3D IC_50_ = 15.07 ± 0.3 µM; 2D IC_50_ = 0.8 ± 0.4 µM; *p < 0.05).

**Discussion:** In conclusion, we propose that the LOT method, despite and because of its simplicity, is a relevant 3D model for drug response measurements that could be scaled up for high throughput screening.

## 1 Introduction

For several years, there has been increasing evidence of the usefulness of three-dimensional (3D) cultures for the study of solid tumors compared to conventional monolayer (2D) cultures. Indeed, differences in transcriptome, proteome, drug response, etc. have been described for several cancer types using different 3D culture methods (Imamura et al., 2015; Bingel et al., 2017; Elia et al., 2017; Wisdom et al., 2018; Thippabhotla et al., 2019). In many cases, these 3D cultures better represent *in vivo* tumors than their 2D counterparts (Weeber et al., 2015; Ganesh et al., 2019), making them more suitable for the description of tumor behavior.

**FIGURE 1:**
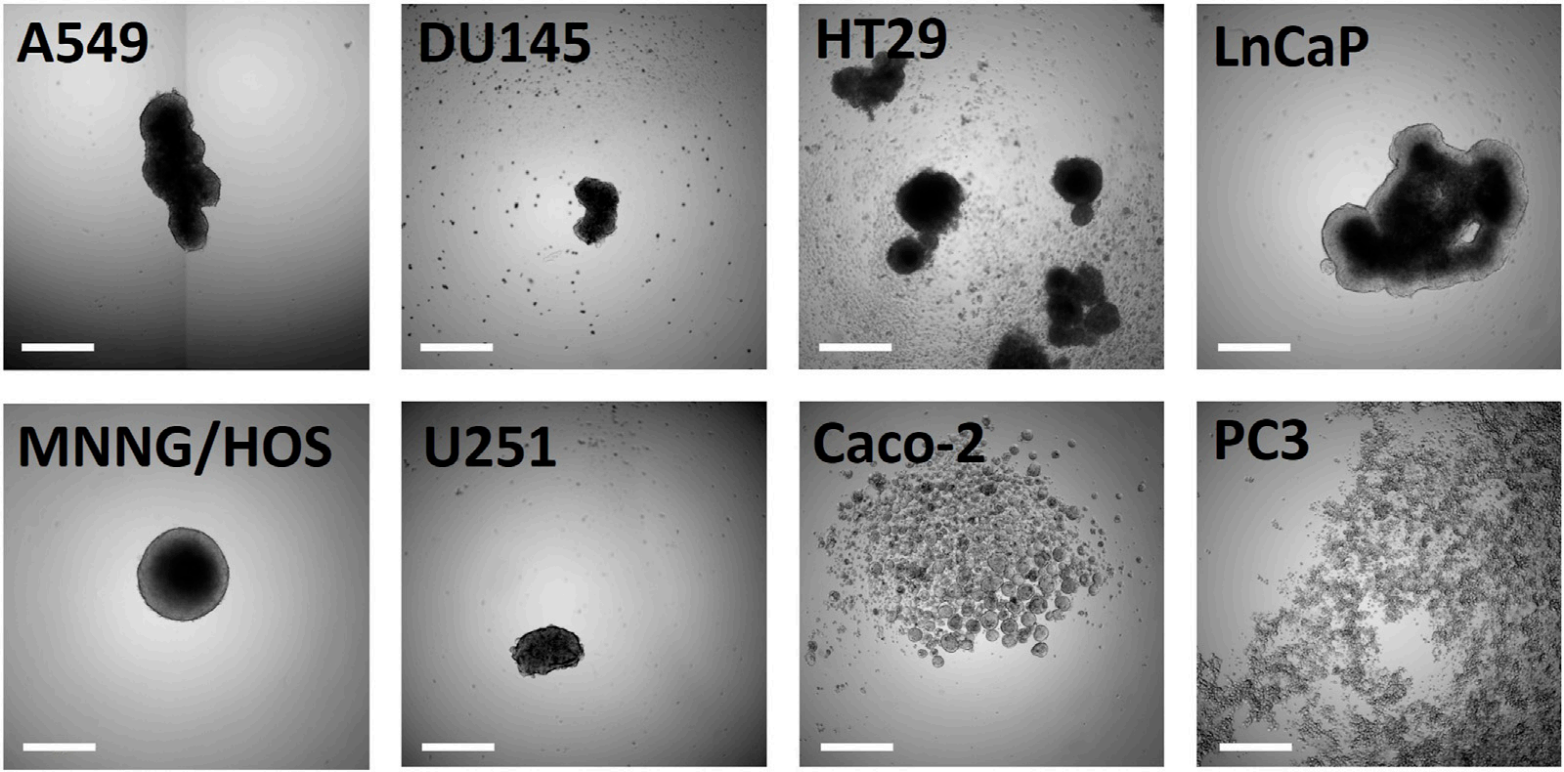
Morphological characterization of cancer cell lines cultured in LOT in flat-bottom 96-well plates at day ten after seeding. Lung adenocarcinoma (A549), prostate adenocarcinoma (DU145, LnCaP, PC3), colorectal adenocarcinoma (Caco-2, HT29), osteosarcoma (MNNG/HOS) and glioblastoma (U251) cell lines were grown for 10 days in 3D. Scale bar corresponds to 500 µm.

Spheroids are one of the most commonly used 3D cell models due to their simplicity. They consist of the aggregation of cells to form a multicellular mass. This spherical organization gives cancer cells properties not observed in 2D, such as the generation of a transport gradient or a stratified structure. Spheroids can be generated using several 3D culture methods (Jubelin et al., 2022), including liquid and scaffold-based methods. Although the first method is rather simple, it may not allow the generation of spheroids (Figure 1). On the other hand, scaffold-based methods may generate spheroids, but they may be dispersed in the matrix, which would increase the time needed to analyze them properly (Imamura et al., 2015). This, in turn, may limit their use in a high-throughput setting.

The production of reproducible spheroids is of particular importance in the context of drug screening. While repeatability is easily achieved in 2D, where all cells are exposed to the same amount of compounds, the transport gradient inherent in 3D culture implies that cells localized to the core of the spheroids may be less exposed to the drug than those on the outer layer. Ensuring that homogeneous spheroids are generated is therefore particularly important, as spheroid morphology can play an important role in treatment response. However, this is often overlooked in studies comparing drug response between 2D and 3D cultures.

State-of-the-art 3D culture methods using bioprinting or microfluidics have been described (Miller et al., 2018; Pinho et al., 2021), but they require very specific equipment, complicate the analysis process and can be difficult to scale up. Therefore, the aim of the present report was to reaffirm the relevance of simpler 3D culture methods to produce highly reproducible spheroids, especially in the context of drug treatment. Two liquid-based cultures were investigated: Liquid Overlay Technique (LOT), which uses culture vessels treated to prevent cells from adhering to their surface, and Hanging Drop (HD), which uses gravity to aggregate cells. Both methods are widely used and therefore well documented. Using LOT and round-bottomed multi-well plates, highly reproducible spheroids of A549 lung adenocarcinoma, LnCaP adenocarcinoma, MNNG/HOS osteosarcoma and U251 glioblastoma cell lines were generated. This method had the advantage of allowing rapid aggregation of cells into spheroids with repeatable morphologies, being inexpensive and implementable in any laboratories. The morphological parameters of the spheroids were easy to assess using microscopic approaches, since the rounded bottom allowed the spheroid to be centered in the well. Finally, this culture model was adapted to drug treatment. But the assessment of the cytotoxicity of the doxorubicin in the 3D specimen required careful selection of the assays. We propose that the LOT method, despite and because of its simplicity, is a relevant 3D model for drug response measurements that could be scaled up for high-throughput screening.

## 2 Material and methods

### 2.1 Cancer cell lines and culture media

The A549 (CCL-185, ATCC) lung adenocarcinoma cell line, the LnCaP (89110211, ECACC, Saliibury, UK) prostate adenocarcinoma cell line, the MNNG/HOS (CRL-1547, ATCC, LGC Molsheim, France) osteosarcoma cell line and the U251 (09063001, Sigma-Aldrich, Saint Quentin Fallavier, France) glioblastoma cell line were cultured at 37°C, 5% CO2 and in an environment saturated in humidity. The A549 were cultured in Ham’s F12-K (21127–022, Gibco) complemented with 10% FBS and 2 mM L-Glutamine. The LnCaP were cultured in RPMI complemented (L0501-500, Dutscher) with 10% FBS, 2 mM L-Glutamine and 1 mM sodium pyruvate. The MNNG/HOS were cultured in DMEM 4.5 g/L D-glucose and 0.11 g/L sodium pyruvate (L0106-500, Dutscher, Bernolsheim, France) supplemented with 5% fetal bovine serum (FBS) (CVFSVF00-01, Eurobio Scientific, Les Ulis, France) and 2 mM L-Glutamine (25030–024, Gibco, Paris, France). The U251 were cultured in DMEM 4.5 g/L D-glucose and 0.11 g/L sodium pyruvate complemented with 10% FBS and 2 mM L-Glutamine. All cell lines were regularly tested for the absence *mycoplasma*.

### 2.2 2D cell cultures

Cell lines were maintained as adherent monolayers in T25 or T75 flasks. When the cells reached 90% of confluency, they were passed following classic cell maintenance protocols. To compare sensitivity to drug treatment, the MNNG/HOS cells were cultured in flat-bottom 96-well adherent plates (3,599, Corning Costar, Boulogne-Billancourt, France).

### 2.3 3D cell cultures

In 3D, spheroids were generated in low-adherence flat- (3,474, Corning Costar) or round-bottom 96-multiwell plates (174926, Thermo Scientific, Saint-Herblain, France), or round-bottom 384-multiwell plates (4,116, Corning Costar) using either the Liquid Overlay Technique (LOT) or the Hanging Drop (HD) method. For cultures using LOT, cells were seeded into low-adherence multiwell plates at a concentration of 20,000 cells per 100 µL or per 50 µL for the 96- and 384-well plates respectively. After 24 h of culture, 50 µL or 25 µL of complete media were added to the wells in the 96- or 384-well plates respectively. Culture media were changed every 2–3 days by replacing 2/3 of the initial volume.

For the cultures using HD, cells were suspended at a concentration of 1 × 10^6^ cells/mL. A methylcellulose (HSC001, R&D System, Abington, UK) solution diluted extemporaneously (1/2) with complete culture medium was then added to the cell suspension to obtain a ratio of 1:4 methylcellulose:suspension (final concentration of methylcellulose: 0.1X). 25 μL droplets of the new suspension were put on the inside of a Petri dish cover. PBS was added to the Petri dish to avoid dehydration of the droplets and the cover was returned to its normal position on the Petri dish. Droplets were incubated in this inversed position for 24 h at 37°C, before being transferred to low-adherence multi-well plates, with one droplet/spheroid per well. Culture medium was added to reach a volume identical to the one for the spheroids generated with LOT.

### 2.4 Cell viability assay

MNNG/HOS cells were treated with increased concentrations of doxorubicin (0.001, 0.01, 0.1, 1, 10, and 100 µM). Cell treatment started 1 day after seeding in either a flat-bottom adherent 96-well plate for 2D culture or a low-adherence round-bottom 96-well plate for 3D culture. After 72 h of incubation with doxorubicin, 5 µL of the supernatant was taken from each well. This supernatant was diluted 1/20 in PBS, and 10 µL of this solution was added to 15 µL of LDH Storage buffer and 25 µL Enzyme Mix from the LDH-Glo™ Cytotoxicity Assay kit (J2380, Promega, Charbonnières-les-Bains, France). Each sample was put in the well of an opaque white 96-well plate. The plate was left at room temperature for 1 h in the dark. Afterward, luminescence rates were assessed using the VICTOR Nivo (Perkin Elmer, Villebon sur Yevette, France) plate reader.

After recovering the samples for the LDH assay, the rest of the supernatant from the treated cells was removed, and the spheroids or cell monolayer were washed and 25 µL of complete medium was added to each well. Then, fluorescent red and green probes from the LIVE/DEAD™ Cell Imaging Kit (Invitrogen™, Whaltam, MA, USA, ref#R37601) were mixed together and 25 µL of this suspension were added to each well. After 15 min of incubation at room temperature in the dark, cells were imaged by fluorescent microcopy using an Operetta CLS High-Content Analysis System (Perkin Elmer).

### 2.5 Microscopic measurement of spheroid morphology

For this study, a spheroid with a satisfactory morphology had to satisfy the following criteria: 1) a minimum diameter greater than or equal to 500 μm, 2) a roundness greater than 0.8, 3) and the presence of a single spheroid per well. Since the diffusion limit of oxygen is 150–200 µm (Olive et al., 1992; Grimes et al., 2014), growing spheroids with a diameter greater than or equal to 500 µm would ensure the generation of a stratified spheroid with a hypoxic core. A geometric shape that can be easily compared between samples is that of the sphere. In addition, a compound would theoretically diffuse more uniformly in a sphere than in any other type of shape. Therefore, a high roundness is an important morphological parameter that the spheroids must have. Roundness is calculated as 4*area/(π*major axis^2^) and a value of 1 corresponds to a perfect disk. Thus, a spheroid with a value of at least 0.8 would closely resemble a sphere. Finally, having of a unique spheroid per well would facilitate the analysis.

The free image analysis software FIJI was used for the measurement of the different parameters. After setting the scale, the parameters “Area,” “Shape descriptors,” “Fit Ellipse” and “Feret’s Diameter” were selected in the “Analyze Particles” dialog box. After the measurement, the values obtained under the labels “Area,” “MinFeret” and “Round” were used to obtain the area of the section, the minimum diameter, and the roundness, respectively. The number of spheroids per well was counted manually.

### 2.6 Statistical analysis

Statistical analyses were performed using GraphPad Prism 7.0 software (GraphPad Software, La Jolla, CA, United States). Significance was determined using a multiple T-test, or a two-way ANOVA test. Error bars show mean ± standard error of the mean. A *p*-value ≤0.05 was considered statistically significant.

## 3 Results

Four cell lines were selected to determine the best 3D culture method for generating spheroids: two cell lines from rare cancers (MNNG/HOS osteosarcoma and U251 glioma cells) and two cell lines from more common cancers (A549 lung adenocarcinoma and LnCaP prostate cancer cells). These cell lines were cultured in 3D using LOT in 96-multiwell plates with flat- or round-bottomed wells for a duration of 20 days. The quality of the spheroids produced was assessed based on their morphological parameters (e.g., area of the spheroid section, minimum diameter, roundness of the sphere, and number of spheroids per well).

**FIGURE 2:**
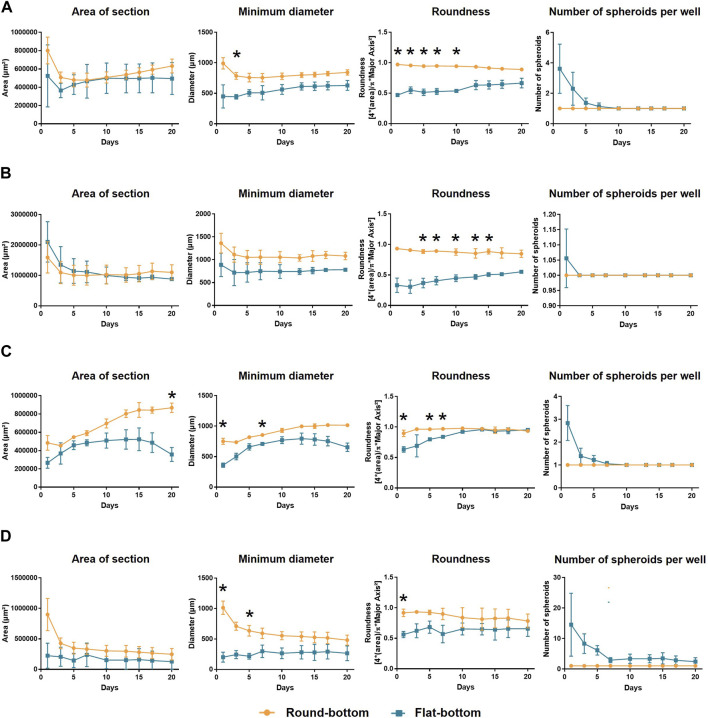
Comparison of the culture plate impact on repeatability of the spheroids formed with LOT. A549 **(A)**, LnCaP **(B)**, MNNG/HOS **(C)** and U251 **(D)** cell lines were seeded either in flat- or round-bottom 96-well plates and were cultured for up to 30 days using LOT. Their morphology (diameter, roundness, number of spheroids) was measured with light microscopy. Statistical test: Two-way Anova with the Geisser-Greenhouse’s and Šídák’s corrections, *p < 0.05.

For all cell lines, except MNNG/HOS at day 20 (*p-value <0.05), the plate type did not have a statistically significant effect on the area of the section (Figure 2). The minimum diameter was significantly increased (*p-value < 0.05) at earlier time points for A549, MNNG/HOS and U251 spheroids grown in round-bottom plates compared to flat-bottom plates spheroids in contrast to LnCaP cells which did not show any significant variation in both conditions (Figures 2A–D). Overall, the spheroids grown in round-bottom plates were slightly larger than those grown in flat-bottom plates and had a minimum diameter of 500 μm at all time points of the experiment. For the four cell lines, round-bottom plates allowed a faster formation of significantly rounder spheroids compared to those produced in flat-bottom plates (*p-value <0.05). Finally, only the round-bottom plates ensured the production of a single spheroid per well from day 1 after the seeding and for the entire duration of the experiment. Taken together, these data showed that the A549, LnCaP, MNNG/HOS and U251 cell lines produced spheroids with highly reproducible morphology when grown by LOT in 96-well round-bottom plates.

**FIGURE 3:**
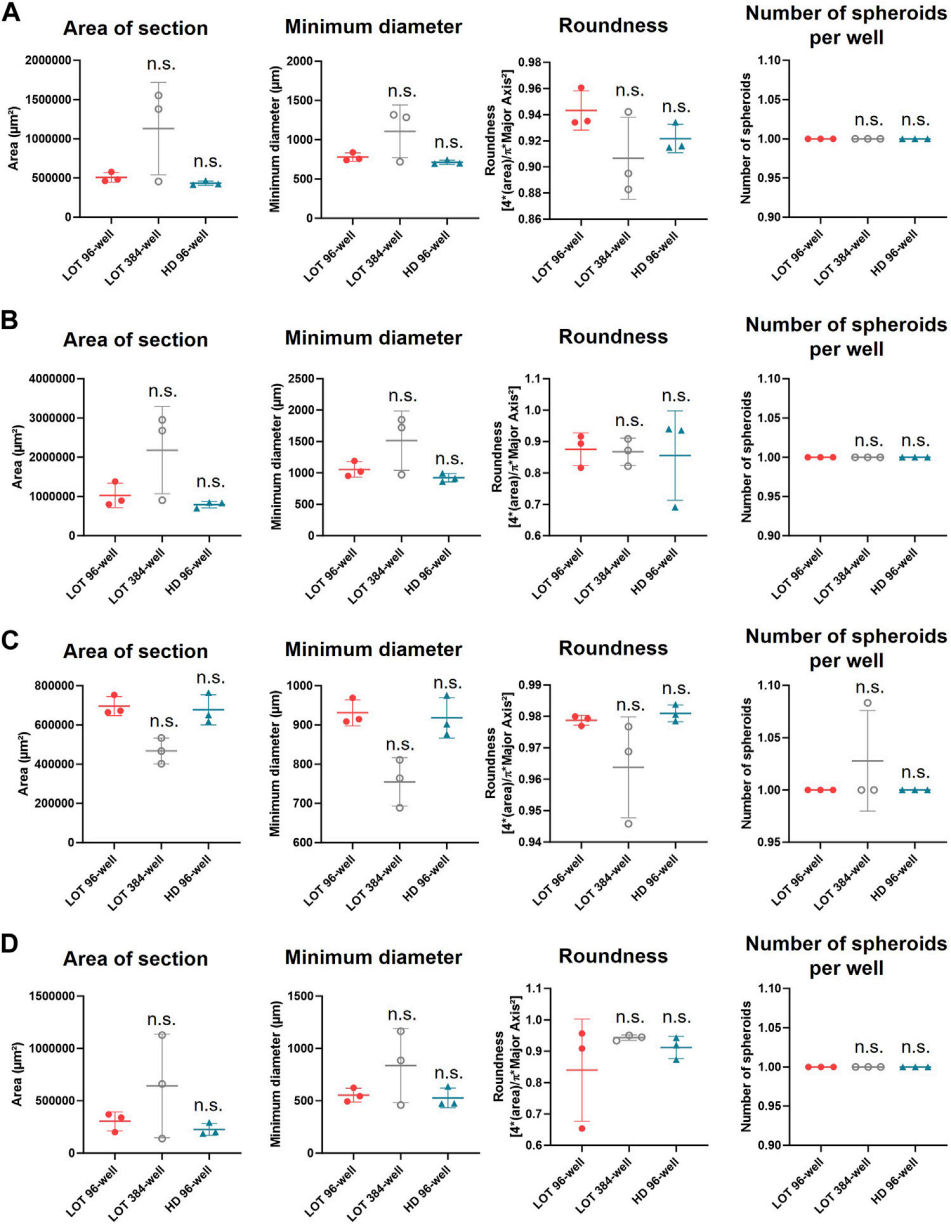
Comparison of the culture volume or the culture method on the morphology of spheroids 10 days after seeding. The morphological characteristics of A549 **(A)**, LnCaP **(B)**, MNNG/HOS **(C)** and U251 **(D)** seeded in round-bottom 96- or 384-well plates and grown with LOT or seeded in round-bottom 96-well plates and grown with HD were measured with light microscopy 10 days after seeding. Statistical test: Mann-Whitney two-sided, comparison with LOT round-bottom condition; n.s., non-significant.

Doubling cell density by using well with a smaller diameter did not further improve the morphological parameters of the four cell lines spheroids when assessed 10 days after seeding. Generally, it even tended to increase heterogeneity between replicates as shown by the higher standard deviation (SD) for the spheroids grown into 384-well round-bottom plates (Figure 3). In all culture vessels assessed, the HD 3D culture method made it possible to generate a single spheroid per well whose morphology validated size and roundness criteria (diameter >500 μm, roundness >0.8) from the first day of culture. However, no significant difference regarding the morphology of the spheroids produced was observed between the spheroids cultured with HD or LOT in round-bottom 96-well plates for all cell lines studied (Figure 3).

To demonstrate the relevance and the efficiency of the LOT in round-bottom plates to evaluate drug response, homogeneous MNNG/HOS spheroids were treated with doxorubicin, and their drug sensitivity was compared to samples grown in 2D. The comparison of cell viability between cells cultured in 2D and in 3D is impaired by the fact that these types of culture lead to highly different cell morphologies and spatial organization. Thus, it is almost impossible to draw definitive conclusions with a sole viability assay, especially if it is an assay originally developed for 2D cultures. In this context, two complementary *in vitro* assays were selected to assess cell viability and response to treatment. MNNG/HOS cells were cultured in 2D or 3D using LOT 96-well plates. Osteosarcoma cells were treated with multiple concentrations of doxorubicin for 72 h. We assessed cell viability based on the culture condition by microscopic observation after LIVE/DEAD™ labelling or by measuring the luminescence rate linked to LDH release from dead cells.

**FIGURE 4:**
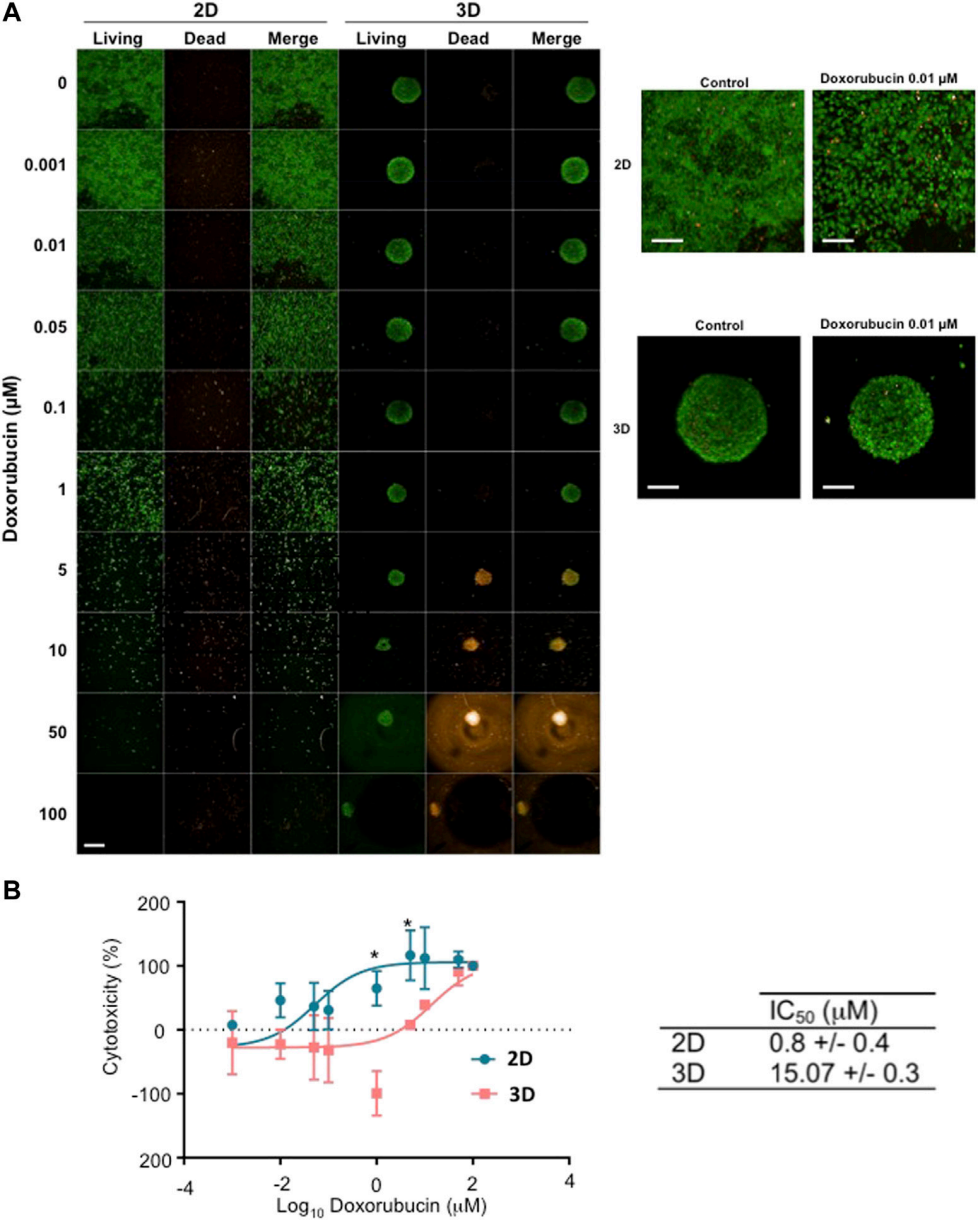
Comparison of the sensitivity to chemotherapy treatment of MNNG/HOS cells grown in 2D or 3D. MNNG/HOS cells were cultured in 2D or 3D in adherent flat-bottom 96-well plates and low-adherent round-bottom 96-well plates respectively. The cell monolayers or spheroids were subjected to various doxorubicin concentrations over 72 h. **(A)** The cell cytotoxicity was measures with LIVE/DEAD™ labelling. Green coloration corresponds to live cells. Orange coloration corresponds to dead cells. Scale bar: 500 µm. **(B)** The dose-response curves were obtained from the test results of the LDH release assay. Statistical test: Two-way Anova with the Šídák’s corrections, *p < 0.05.

While the LIVE/DEAD™ assay is very useful to assess cell viability in 2D, it lacks resolution in a 3D setting if the imaged specimens are too thick. However, here it allowed to reveal changes in the shape of the cells depending on the concentration of doxorubicin used (Figure 4A). Both in 2D and 3D, we observed an increase in cell size from the concentration 0.01 µM. In 3D, the cells started to shrink again at 1 μm, and this coincided with a decrease in the spheroid diameter. 100 μM of doxorubicin resulted in the disintegration of the spheroids (Figure 4A).

Although these microscopic observations provide simple and straightforward insights, they are not conclusive in the case of 3D culture. Therefore, an additional assay was added to allow for an unbiased comparison between 2D and 3D. The LDH release assay was performed on the supernatant of the cells previously imaged (Figure 4B). The dose-response curves of doxorubicin were different in 2D compared to 3D cell cultures. For the concentrations of 1 µM and 5 μM, a significant decrease in cytotoxicity was observed for the 3D culture condition. This translated into an increased IC_50_ in 3D (3D IC_50_ = 15.07 ± 0.3 µM), which was 18.8 times higher than the IC_50_ in 2D cell cultures (2D IC_50_ = 0.8 ± 0.4 µM) (Figure 4, *p < 0.05). Overall, the present data showed decreased sensitivity of MNNG/HOS cells to doxorubicin when cultured in 3D rather than in 2D.

## 4 Discussion

The ability to form spheroids is not uniform among cell lines, as shown in Figure 1. When grown by the LOT method, the formation of spheroids depends on the intrinsic ability of the cells to aggregate. Modifying the media and/or adding matrix could improve spheroid aggregation. For example, when attempting to grow PC3 cells in 3D using the same medium as in 2D, we did not observe spheroid formation in our LOT condition (Figure 1). However, other studies were able to produce round PC3 spheroids with a defined border by growing them with Matrigel™ (Härmä et al., 2010). In addition, several 3D culture media formulations have been described for various application (Fleurence et al., 2016; Gheytanchi et al., 2021). In the context of comparing drug sensitivity between 2D and 3D cultures, it was essential to reduce variations in the parameters tested. The decision was made to grow each cell type in 3D with the corresponding medium traditionally used in 2D. This would also enable any laboratory to transition to 3D culture using the same medium they already use for their monolayer cultures. As a results, the primary focus was on modifying the 3D culture method to enhance spheroid generation.

The spatial organization of spheroids has a direct impact on both molecular diffusion and cell imaging. It is essential that the spheroids have a homogeneous morphology if we want the observed results to be significant. Most biochemical assays commonly used in research have been originally designed and optimized for 2D cell cultures. Adapting these assays to 3D culture models often requires protocol optimization, as the spatial organization of the spheroids plays a direct role in the diffusion of molecules. By ensuring the generation of spheroids w size aith a consistent size and shape, it is possible to reduce the influence of morphological variability on the significance of the results obtained.

Spheroid morphology was assessed by measuring several shape parameters: area of the cross-section and minimum diameter to translate spheroid size, roundness to describe the spheroid shape, and number of spheroids per well. Three main validation criteria for the 3D culture method were selected in this study: 1) the spheroids had to have a diameter greater than or equal to 500 µm in order to generate a hypoxic core; 2) the spheroids had to have a shape similar to that of a sphere to ensure that the diffusion of the molecules inside the spheroid was homogeneous between conditions; 3) the 3D culture methods had to generate a single spheroid per well. The 3D culture method that allowed the formation of spheroids meeting all these criteria was LOT in round-bottom 96-well plates for all four cell lines studied (lung adenocarcinoma A549, prostate adenocarcinoma LnCaP, osteosarcoma MMNG/HOS, glioblastoma U251). In addition, the round-bottom plate facilitated image acquisition as the spheroid was always in the center of the well due to gravity. The formation of morphologically homogeneous spheroids across wells appeared to be dependent on the aggregation rate. The round-bottom plates allowed the generation of highly repeatable spheroids by increasing cell-cell contacts.

The aim of this study was to provide a simple 3D culture method of the A549, LnCaP, MNNG/HOS and U251 cell lines, for further characterization and use of the spheroids generated to study mechanisms involved in cancer initiation, progression and treatment response. As a proof of concept, the response of MNNG/HOS grown in 3D with LOT in low-adherence 96-well plates to the conventional chemotherapy doxorubicin was measured and compared to 2D culture. The study of drug efficacy in 3D is different from that in 2D. In fact, the spatial organization of the spheroid limits the type of assay that can be used. Indeed, most fluorescent cytotoxic assays on living spheroids would be compromised by the thickness of the sample. A more suitable live assay would require the use of spheroid supernatant, such as the LDH release assay. Alternatively, fixation and clarification or sectioning of the spheroids would be required to image the inside of the sample.

Similar to what has already been observed in studies covering other cell lines (Imamura et al., 2015), we describe a decrease in the drug sensitivity of MNNG/HOS cells cultured in 3D compared to 2D. This difference in sensitivity may have multiple explanations. The presence of a diffusion gradient, hypoxic conditions and increased cell-to-cell and cell-to-extracellular matrix (ECM) interactions are all molecular cues that can affect the activation of the signaling pathways, biological processes, and gene and protein expressions involved in drug resistance (Zschenker et al., 2012; Gangadhara et al., 2016; Nath and Devi, 2016; Bingel et al., 2017).

The spatial organization of spheroids can also limit treatment efficacy. The presence of a diffusion gradient applies not only to oxygen, but also affects the distribution of chemotherapy molecules. In contrast to 2D cultures, where the doxorubicin is evenly distributed among all cells, the drug may have difficulty reaching the more central cells of the spheroids (Ong et al., 2010; Ma et al., 2012). Usually, larger and more compact spheroids tend to be less treatment-sensitive (Däster et al., 2016; Gencoglu et al., 2018; Thakuri et al., 2019). In the present study, we treated “young” spheroids. We made this choice to minimize the difference in cell number between 2D and 3D cultures, since cell proliferation typically slow down significantly in 3D. It is therefore possible that treating older spheroids could result in an even greater loss of sensitivity than that observed here, as the spheroids would have had more time to compact and establish cellular interactions with their neighboring cells. Hypoxia, which can occur in large spheroids, may also be associated with decreased drug sensitivity. Hypoxia could lead to greater cancer resistance to treatment by promoting the expression of an efflux pump on the cell surface, thereby inducing an anti-apoptotic effect, by promoting genomic instability and slowing cell proliferation (Rohwer and Cramer, 2011). Finally, numerous chemotherapeutic molecules, such as doxorubicin, target cancer cells with a fast proliferation rate. As a result, drugs targeting fast-proliferating cancer cells may prove ineffective against the slowed or arrested proliferation observed in spheroids. (Imamura et al., 2015).

In conclusion, we show here that the culture of spheroids in round-bottom multiwell plates using LOT allowed the production of repeatable spheroids for multiple cell lines. This culture method has the advantage of being easy to implement by any laboratory. It is also inexpensive and can be scaled up for high-throughput screening. Although spheroid cultures require optimization of the analysis methods developed for 2D cultures, we were able to show that the efficacy of a treatment can be measured by multiple assays. For all these reasons, the LOT method is a relevant 3D model and we recommend its implementation for any drug screening study.

## Data Availability

The original contributions presented in the study are included in the article, further inquiries can be directed to the corresponding author.
